# A STD-NMR Study of the Interaction of the *Anabaena* Ferredoxin-NADP^+^ Reductase with the Coenzyme

**DOI:** 10.3390/molecules19010672

**Published:** 2014-01-07

**Authors:** Lara V. Antonini, José R. Peregrina, Jesús Angulo, Milagros Medina, Pedro M. Nieto

**Affiliations:** 1 Instituto de Investigaciones Químicas, CSIC, Americo Vespucio, 49, Sevilla 41092, Spain; E-Mail: laravaantonini@yahoo.com.ar; 2 Departamento de Bioquimica y Biologia Molecular y Celular, Facultad de Ciencias and Institute of Biocomputation and Physics of Complex Systems (BIFI), Universidad de Zaragoza, Zaragoza 50009, Spain; E-Mails: jrp8@st-andrews.ac.uk (J.R.P.); mmedina@unizar.es (M.M.)

**Keywords:** saturation transfer difference NMR spectroscopy, flavoenzymes, hydride transfer, isoalloxazine-nicotinamide interactions, CORCEMA-ST

## Abstract

Ferredoxin-NADP^+^ reductase (FNR) catalyzes the electron transfer from ferredoxin to NADP^+^ via its flavin FAD cofactor. To get further insights in the architecture of the transient complexes produced during the hydride transfer event between the enzyme and the NADP^+^ coenzyme we have applied NMR spectroscopy using Saturation Transfer Difference (STD) techniques to analyze the interaction between FNR_ox_ and the oxidized state of its NADP^+^ coenzyme. We have found that STD NMR, together with the use of selected mutations on FNR and of the non-FNR reacting coenzyme analogue NAD^+^, are appropriate tools to provide further information about the the interaction epitope.

## 1. Introduction

The main role of plastidic ferredoxin-NADP^+^ reductases (FNRs) (EC 1.18.1.2) is the photosynthetic transfer of reduction equivalents from ferredoxin (Fd) to NADP^+^ via its FAD cofactor. *In vivo* this process is reversible, and thus FNR can also provide electrons to different proteins using NADPH as reducing agent [1,2]. Moreover, FNR is highly specific for NADP^+^/H *vs.* NAD^+^/H:

2Fd_red_ + NADP^+^ ⇆ 2Fd_ox_ + NADPH

Plastidic FNR fold in two domains; one contains the non-covalently bound flavin adenine dinucleotide (FAD) cofactor and other binds the NADP^+^ coenzyme. In *Anabaena* FNR the FAD-binding domain is made up of six antiparallel *β*-strands disposed in two *β*-sheets and includes residues 1–138. The NADP^+^-binding domain corresponds to the residues 139–303 and comprises seven *α*-helices packed against a core of five parallel *β*-strands. The FAD cofactor is in an extended conformation with its reactive isoalloxazine ring sitting at the interphase between the two domains. Several residues at the NADP^+^ binding domain have been reported to determine coenzyme binding, specificity and enzymatic efficiency in FNR [3,4,5,6,7,8]. One of the most characteristic structural features at the NADP^+^ binding domain in plastidic FNRs is the conservation of a C-terminal Tyr that stabilises the *re*-face of the isoalloxazine through p-p stacking, occupying the putative nicotinamide catalytic binding site in the free enzyme. This Tyr plays a key role in modulating the entrance and binding of the nicotinamide portion of the coenzyme in a catalytic competent orientation into the active site [6,9,10,11]. Recent theoretical studies suggest that upon coenzyme binding this side-chain gets slightly displaced, remaining as part of the active site to favour the optimal geometry between the nicotatinamide and isoalloxazine rings for HT while preventing formation of a too strong ionic pair between both rings incompatible with an efficient turnover and with the reversibility of the process [12,13,14]. This residue also contributes to stabilize the flavin semiquinone and to set the flavin midpoint-reduction potentials, therefore, modulating the electron transfer rates with the protein partners [6,15]. The single Y303S mutation has been shown to significantly enhance specificity for NAD^+^. Combined mutations have also been produced at the NADP^+^ binding domain regions involved in coenzyme binding in attempts to change the FNR specificity for the coenzyme [16]. Combination of mutations at the pyrophosphate, 2'-phosphate regions, and including Y303S as in the T155G/A160T/L263P/Y303S (PP3CT) mutant with extended binding pockets for both nucleotides, did not improve activity with NAD^+^, despite structures of these FNRs show how particular coenzyme-binding regions resembled motifs found in NAD^+^/H-dependent enzymes of the FNR family [16].

In the present study we describe the application of the saturation transfer difference spectroscopy (STD NMR) [17] technique to further gain insights into the architecture of the FNR:coenzyme interaction during the hydride transfer reaction. Although NMR studies have been published on this system, they correspond to a complete structural elucidation [18,19]. In this methodology the protein signals are observed and analyzed to obtain restrains to calculate the complete structure of a stable complex. On the contrary, STD analyzes the signals of the excess of ligand in fast equilibrium between the bound and free states (see Supplementary Information). While in the case of the complete structure determination isotopic labeling of the protein is mandatory for a protein of this size, the STD experiment allows the use of non-labeled samples. However, a tentative structure is needed to predict the values of the STD and extract structural information. Since 1999, STD NMR has become one of the most powerful and versatile NMR techniques for observing ligands, with growing applications both in academic research and in the pharmaceutical industry as a basic method in drug discovery, design, and optimization [18,20,21,22,23].

**Figure 1:**
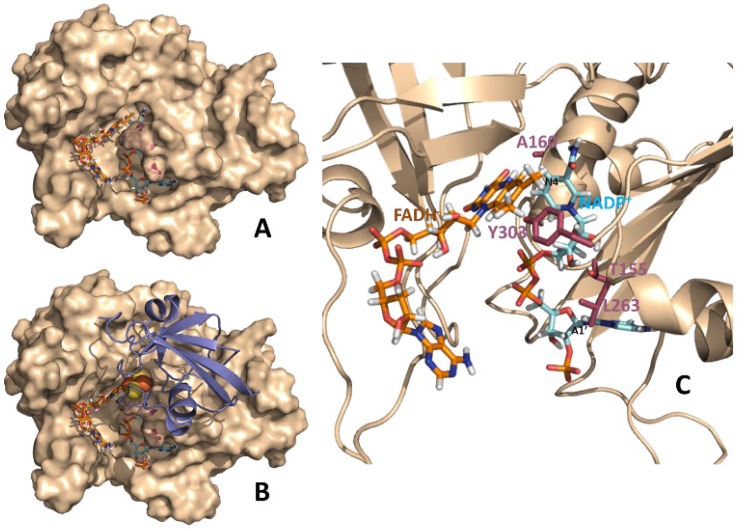
Theoretical models for binary FNR:NADP^+^ and ternary Fd:FNR:NADP^+^ complexes from *Anabaena*. (**A**) Structural model at the equilibrium of the molecular dynamics simulations of a catalytically competent complex between reduced FNR and NADP^+^ [14]. (**B**) Structural model for the Fd:FNR:NADP^+^ ternary complex. The model has been constructed by structural alignment of the equilibrium molecular dynamics FNR:NADP^+^ complex (**A**) with the crystal structure of the Fd:FNR complex (PDB 1ewy) [24]. **C**. Detail of the relative disposition between the NADP^+^ coenzyme and the FAD cofactor in the binary FNR:NADP^+^ complex. The polypeptide chain of FNR is shown in wheat (in **A** and **B** as surface and in **C** as cartoon). FAD and NADP^+^ are represented in sticks with carbons in orange and blue, respectively. N4 and A1’ positions of NADP^+^ are black labeled to help orientating the molecule. In **B** Fd is shown in violet cartoon with its iron-sulphur cluster in spheres. Residues mutated in this work are highlighted in raspberry either as surface dots (**A** and **B**) or CPK sticks (**C**).

In this technique, the saturation from the complex is detected in the average ligand signals, by difference between the reference and the saturated spectra. The proper analysis of STD data can afford a diversity of information about the local structure of transient biomolecular complexes. Initial qualitative applications of STD-NMR include the screening of small ligand libraries, or mapping of theirs binding epitopes by comparison of relative STD intensities. Further semiquantitative applications assumed that the relative intensities of different signals do not depend on the saturation time, which is not true. Therefore, an accurate quantification requires cancellation of the effects of relaxation and/or rebinding on the STD build-up, which applies at the limit of zero saturation time (yielding the initial slope of the STD amplification factor, STD-AF_0_). More precise analysis can be achieved by comparing experimental and theoretical STD-AF_0_ values, leading to the possibility of deducing more precise geometrical information. Although this method suffers from the need for preliminary structural models to calculate theoretical values by CORCEMA, it has been used satisfactorily in several cases, including examples of multiple binding modes.The interaction between FNR and the coenzyme has been described as a multistep mechanism with two different detectable charge transfer intermediates that are inaccessible for the NMR time scale, as it is a too slow spectroscopic technique for the detection of short-lived species [25]. Nevertheless, the capacity of NMR for obtaining average observables from highly dynamic situations can be used for extracting information on the geometry of the complexes. In order to have a steady state system stable enough for the application of NMR techniques we have studied the system with the oxidized forms of the FAD cofactor and the NAD(P)^+^ coenzyme. This situation, analogue to all the crystallographic complexes so far reported for this system (Figure 1), provide advantageous information in solution. To fully explore the performance of the technique we have used the WT *Anabaena* FNR and the Y303S and PP3CT mutants. In this way we used three FNR variants with different binding constants for NADP^+^ and NAD^+^ [16].

## 2. Results and Discussion

### 2.1. STD NMR Experiments with NADP^+^ and NAD^+^

**Figure 2:**
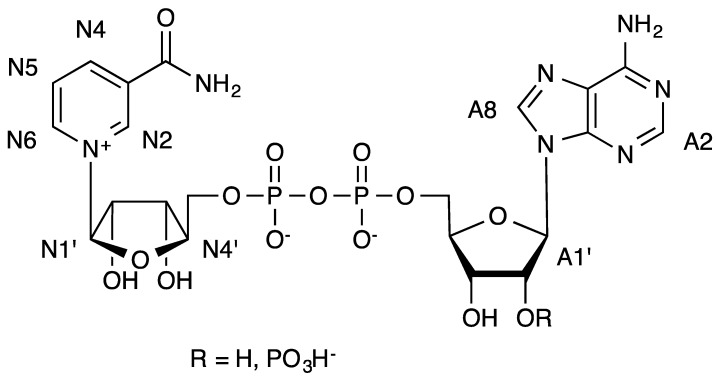
Formulae of NAD^+^ (R = H) and NADP^+^ (R = PO_3_H^−^) and numbering used in the text.

We have performed a complete NMR study based mainly in the application of STD NMR techniques [18,21,24] to the complexes formed between WT *Anabaena* FNR, and two of its previously characterized mutants T155G/A160T/L263P/Y303S (PP3CT) and Y303S, with the protein natural coenzyme, NADP^+^, or with its analogue NAD^+^ (Figure 2, see Supplementary Information for an example of an STD experiment) [26].

In all cases the proton that received more magnetization was the proton at position A2 of the adenosine. In Table 1, the absolute fractional STD values [27] for the proton 2 of adenosine at 2 s of saturation time are compared for all the complexes. These values are usually interpreted in terms of difference of affinity of the protein mutants for the ligands NADP^+^ and NAD^+^ [28,29]. However, it should be reminded that STD is a magnitude derived from binding kinetics and the later assumption is true only when k_on_ are similar, often when the association is controlled by the diffusion limit [30]. In this case, the differences on the accessibility of the ligands to the binding site should be different due to the FNR mutations. Therefore, this reasoning should be taken into account as a lower value of absolute STD does not reflect necessarily a lower affinity, and it can be the consequence of an unfavorable value of k_on_ and/or k_off_. For example excessively high k_on_ would lead to a lower normalized STD [31,32].

**Table 1:** Absolute fractional values of STD (*η_STD_* = *I_0_ − I_sat_/I* × *100*) for the adenosine proton A2, taken at saturation time of 2s for the complexes between FNR, PP3CT and FNR: Y303S with NADP^+^ and NAD^+^.

FNR variant	WT-NADP^+^	WT-NAD^+^	PP3CT-NADP^+^	PP3CT-NAD^+^	Y303S-NADP^+^	Y303S-NAD^+^
A2	30	10	1.5	2.6	7.4	6.2

From the whole study two cases where the complete procedure for the analysis of STD could not be used have appeared. One was the case of WT FNR in complex with NAD^+^; where a complete analysis based on the method of the initial slopes of the STD_0_ [30] the most precise, was unfeasible as the coenzyme decomposed. Additionally the STD peaks at short saturation times for the complexes of PP3CT and Y303S with NADP^+^ were too weak, due to unfavorable kinetics, and could not be accurately integrated. In these two cases we have used the STD NMR relative values at 2 s. for epitope mapping (Table 2). Those, however, do not exhibit the characteristic behavior caused by differential relaxation of aromatic—aliphatic protons that biased the results at long saturation times increasing the aromatic STD prevalence in the epitope mapping.

**Table 2:** STD relative values at 2 s saturation time for Saturation Transfer Experiments, normalized (100%) against the larger absolute STD fractional value for each ligand, performed for the complexes between WT, PP3CT and Y303S *Anabaena* FNR variants with NADP^+^ and NAD^+^.

FNR variant	WT-NADP^+^	WT-NAD^+^	PP3CT-NADP^+^	PP3CT-NAD^+^	Y303S-NADP^+^	Y303S-NAD^+^
A2	100	100	100	100	100	100
A8	13	16	27	29	12	23
A1’	43	40	40	50	-	-
N2	17	-	27	19	17	19
N4	43	-	40	35	44	32
N5	30	-	40	-	35	27
N6	13	-	26	19	15	21
N1’	17	-	27	21	-	-
N4’	20	-	40	33	-	-

#### 2.1.1. WT *Anabaena* FNR

STD experiments at growing saturation times were obtained for the complex of FNR_ox_ with NADP^+^. The results were consistent with the binding of NADP^+^ in an extended conformation where the whole chain of the dinucleotide has important interactions at both the aromatic moieties (adenine and nicotinamide) and at the two riboses. The largest STD was observed for the A2 proton of adenine indicating that this part of the NADP^+^ has a close contact with the protein. However, in this case the nicotinamide showed also strong STD signals (Table 3).

**Table 3:** STD initial growing rate relative values for Saturation Transfer Experiments, normalized against the larger STD initial growing rate (100%), performed for the complexes between WT *Anabaena* FNR, PP3CT and FNR:Y303S with NADP^+^ and NAD^+^.

	FNR-NADP^+^	FNR-NAD^+^	PP3CT-NADP^+^	PP3CT-NAD^+^	Y303S-NADP^+^	Y303S-NAD^+^
A2	100		100	100		100
A8	15		49	57		17
A1’	46		66	48		34
N2	17		50	31		20
N4	38		53	39		44
N5	33		52	-		42
N6	14		76	41		18
N1’	27		54	34		-
N4’	20		68	51		26

When we analyzed the interaction of FNR with NAD^+^, peaks arising from the adenosine mononucleotide part were clearly detected with medium intensity, absolute STD value of 10.0 (see Table 1). Intriguingly, the nicotinamide nucleotide was not showing STD peaks at all. We attributed this behavior to the interaction of only half of the dinucleotide with FNR while the nicotinamide is folded back outwards, instead of interacting with the other sub-binding site. The lack of previous data respect to this interaction could be due to the earliest measurement by UV of the interaction between FNR and nucleotides that detected the changes in the UV spectra caused by the π-π interaction between the nicotinamide and the isoalloxazine from the coenzyme rings that would not be present in such complex. A crystallographic structure (pdb: 1quf) where the conformation of the dinucleotide was folded back out from the side occupied by the nicotinamide ring of the NADP^+^ support this observation. Unfortunately, NAD^+^ decomposed in presence of FNR avoiding collection of further data at different saturation times. Therefore we used for comparison the relative STD values at 2 s that are shown Table 2. Comparing both nucleotides the STD data were consistent with a participation of both to the binding to NADP^+^ and only the adenosine one in the case of NAD^+^.

#### 2.1.2. T155G/A160T/L263P/Y303SPP3CT (PP3CT)

Secondly, STD experiments were recorded with a FNR tetramutant and both dinucleotides. As consequence of the mutations the PP3CT variant has extended cavities for the binding sites of both nucleotides conducting to lower values of the binding constants that were decreased to less than 1 μM for NADP^+^ and 1.341 mM for NAD^+^ [26]. Interestingly those were at the upper and lower limits of the range of binding constants for the optimum STD performance. Thus, in order to perform a similar STD NMR analysis, we increased the ligand concentration until 5 mM to obtain reasonable signal to noise ratio. The binding epitope has similar characteristics as the FNR:NADP^+^ one, being the A2 protons those that received the larger saturation (Table 3). Interestingly, NAD^+^ did interact with the PP3CT along all its length including with the PP3CT nicotinamide-binding site. In this pair both complexes had low values of STD, reflecting that low STD values besides by a very weak interaction as in PP3CT NAD^+^ with a *K*_d_ in the mM range can be caused also by a very strong interaction, low μM as PP3CT NADP^+^ due a low rate of exchange between the bound and free forms of the ligand.

The relative STD_0_ for the nicotinamide ring for proton N6 for the complex between PP3CT and NADP^+^ displays a value of 76% while in the case of FNR was 14%. This difference is relevant and might be reflecting the Y303S mutation that allows the nicotinamide ring to stack to the isoalloxazine ring or the flip over deepening H6 into the groove.

#### 2.1.3. FNR Y303S

The Y303S FNR complexes with NADP^+^ and NAD^+^ were also examined by means of STD NMR experiments. The complex with NADP^+^ displayed a very poor signal to noise ratio at sort saturation times, preventing the accurate construction of a saturation curve and, therefore, to apply the STD initial growing rate procedure. In this case, the failure to obtain enough amount of saturation transferred at short saturation times, even after the increase of the ligand concentration until 5.0 mM, is probably due to the low dissociation constant reported for the complex, lower than 0.01 μM [6]. Consequently, we compared the binding epitopes calculated at 2 s. saturation time (Table 2). As in the case of the PP3CT complexes the binding epitope covers the complete extent of both coenzymes. This additional stabilization has been reported for mutations in this residue with non-aromatic aminoacids. It was suggested to be caused by an additional aromatic-aromatic interaction between the isoalloxazine moiety and the nicotinamide ring that impairs the physiological hydride transfer in the catalytic pair prevented by the presence of Tyr303 between the nicotinamide and the isoalloxazine rings. This effect, initially attributed to the rupture of the π-π interaction between the aromatic rings, has been recent revised using theoretical studies that have provided arguments in favor to the loss of electrostatic interaction due to the larger distance between the NADP^+^ and FADH^−^.

### 2.2. CORCEMA Analysis of STD

The analysis of the bound ligand structures from STD NMR data could be done by quantitative analysis of the predicted STD value. CORCEMA-ST calculates the theoretical STD through the complete analysis of the relaxation matrix based on the three-dimensional structure of the complex, association and dissociation constants, and other NMR parameters [33]. These theoretical values can be compared against the experimental ones using the factor R-NOE between the experimental and the theoretical values.

#### WT *Anabaena* FNR

Three models were evaluated for comparison with the experimental values: (a) a crystallographic structure (PDB 1gjr) [3], with FAD and NADP^+^ both in the oxidized form; (b) the final molecular dynamics snapshot obtained from extensive molecular dynamic calculations corresponding to the physiological pair, oxidized FAD and NADPH [14]; and (c) FAD and NADP^+^ both in the oxidized state obtained from a molecular dynamics snapshot, replacing NADPH for NADP^+^ by adding the hydride, and further minimized.

**Figure 3:**
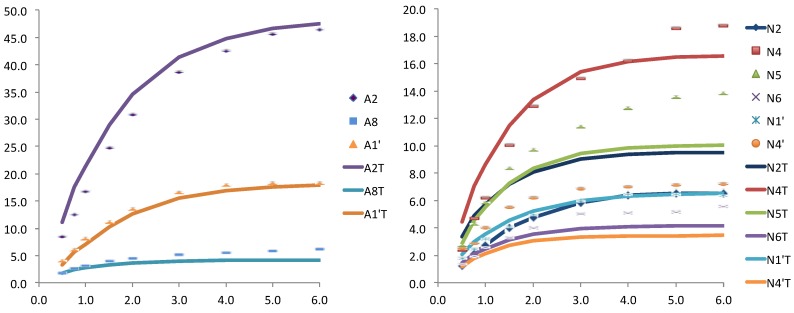
Relative STD experimental for WT *Anabaena* FNR and NADP^+^ (dots) and theoretically predicted (lines) with CORCEMA for the crystallographic structure (PDB: 1gjr), for the adenosine (left) and for the nicotinamide (right) nucleotides of the coenzyme.

The results for the first pair (case a) were very reasonable, in particular those related to the adenosine nucleotide showing an excellent agreement (see Figure 3). The nicotinamide moiety showed worse results, probably as consequence of its larger flexibility. This flexibility is in agreement with the structures obtained for the pair FAD and NAD^+^. Where the nicotinamide nucleotide did not received saturation from the enzyme, as these structures were folded back out of the binding site.

**Figure 4:**
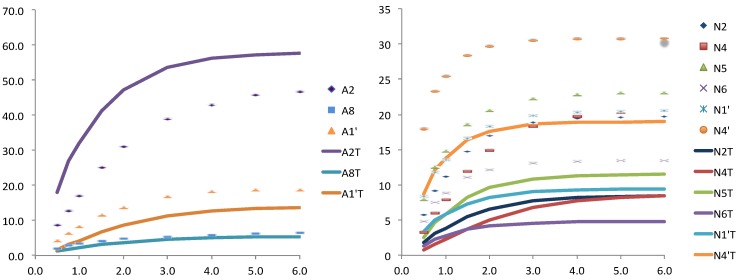
Relative STD experimental (dots) and theoretically predicted (lines) with CORCEMA for one structure taken from MD simulation and further minimized, for the adenosine (left) and for the nicotinamide (right) nucleotides.

On the contrary the theoretical results obtained for an equilibrated snapshot of the molecular dynamics (case b) were clearly far from the convergence with the experimental ones. This is not surprising if we consider that these are instantaneous structures that contribute to the averaged situation but do not represent a steady state position. Finally, when the NADPH from the MD structure was manually reduced by adding a hydride, and subject to minimization the fitting was improved, especially for the adenosine moiety (Figure 4).

### 2.3. Discussion

Interestingly, most of the binding epitopes calculated from STD-NMR converge to the same binding mode that is compatible with an extended conformation of the dinucleotide along a cleft parallel to the interphase between the two domains of FNR. However, the absolute STD values are very dispersed, reflecting the broad range on affinity constants. In all these cases the binding epitopes were very similar being A2 protons from the adenosine ring those with the 100% relative STD.

According to our analysis based in STD, the complex between FNR and NADP^+^ is consistent with the crystallographic structure for the same pair of oxidized coenzymes. The back-calculated STD values obtained by applying CORCEMA-ST to the crystallographic structure (pdb: 1grj) give very good results, in particular for the adenosine nucleotide. In clear contradiction, the complex between FNR and NAD^+^ showed a different epitope compatible with the single interaction with the adenosine nucleotide in its binding pocket but not from the nicotinamide ring. In fact, the measurement of binding using the standard UV method did not yield prove of binding due to the absence of interaction with the nicotinamide aromatic ring. A crystallographic complex, 1quf, obtained by soaking showed a structure that could explain these results. In this structure the nicotinamide nucleotide is folded back out from the cleft.

The tetramutant PP3CT STD-NMR showed very weak transferred peaks in both complexes. While in the case of the NADP^+^ the low value can be attributed to the strong affinity that decrease the exchange rate between the cofactor bound and free in solution, in the case of the NAD^+^ is due to the low affinity, in the limits of the detection of this technique. These hypotheses were confirmed by increasing the concentration of ligand in the experiments obtaining larger reasonable values of absolute STD.

The case of the FNR Y303S was similar to the PP3CT being the affinity for NADP^+^ too strong for completion of the binding curve and only saturation times longer than 2 s. or larger were measurable. On the other hand the NAD^+^ was still on the reasonable kinetic window for the application of STD. The STD results from the PP3CT:NAD^+^ are in clear agreement with the interaction mode established for FNR WT-NADP^+^, extended mode with both nucleotides interacting with the protein in a large cleft with two sites for each nucleotide.

Interestingly, the range of *K*_d_ for some of these complexes were far from the limits established for their detection using STD. We assume that due to the nature of the reaction, hydride transference related to photosynthetic reactions, one of the fastest in the nature the accumulation of free ligand previously bounded is enough to be observed.

## 3. Experimental Section

Dinucleotides were purchased from Aldrich (Sigma-Aldrich Quimica SL, Madrid, Spain) and used without further purification. Protein samples were prepared as described previously [6,26]. NMR Experiment were performed in a Bruker AVANCE 500-MHz spectrometer (Bruker, Wissembourg, France) equipped with inverse triple resonance probe TXI using samples of 500 μL for STD with 1–5 mM ligand concentration and 40–60 μM protein concentration, corresponding to ratios protein/ligand from 1/200 to 1/40. All samples were prepared in buffer TRIS d11 15 mM at pH 8 in D_2_O and were exchanged several times with the same buffer using Centricon^®^ filters (Millipore, EMD Millipore Corporation, Billerica, MA, USA) with cut-off of 30 kDa.

STD experiments were performed using the standard sequence at 288 K using a train of gaussian pulses of 49 ms and 10–60 Hz power spaced by a delay of 1 ms. for selective saturation at 40 ppm (off-resonance) and 0.75 (on-resonance) recorded in an interleaved acquisition. Experiments without protein were performed in order to ensure the absence of direct irradiation in the experimental conditions. Saturation times used for the construction of the growth curves were 0.5, 0.75, 1.0, 1.5, 2.0, 3.0, 4.0, 5.0, 6.0 s to obtain the STD saturation curves. Theoretical calculations of values of STD were performed using CORCEMA-ST.

The binding epitope was characterized by the analysis of initial slopes of the STD intensities at 25 °C [27]: the experimental (I_0_ − I_sat_/I_0_) curves were fitted to an exponential function described by the equation: STD (t_sat_) = STD_max_ (1 − e ^−ksat·tsat^), which allows to calculate STD at zero saturation time (initial slopes) by multiplying the resulting parameters STD_max_ and k_sat_ [34]. The epitope is obtained by normalization of the whole set of initial slopes against the highest value, and expressing the result in percentage.

The Cartesian coordinates of the crystal structure of the complex were employed for the full relaxation matrix calculations. The crystallographic structures were prepared by inserting hydrogen atoms into the structure according to available routines in Maestro. As no chemical shift assignment of the protein protons was available, they were predicted by using the program SHIFTX [35]. All exchangeable hydrogen atoms were excluded in the calculations, as the STD NMR experiments were performed in D_2_O. We assumed that pdb coordinates for the bound and free protein were identical and several cycles were performed to reach the optimized parameters. To reduce the dimensions of the matrices, a cut off of 8 Å from the ligand was used. The STD intensities for each binding mode were calculated as percentage fractional intensity changes, **S**_calc,k_, from the intensity matrix **I(t)** (**S**_calc,k_ = ([(**I_0k_** − **I(t)_k_**) × 100]/**I_0k_**), where **k** is a particular proton in the complex, and **I_0k_** its thermal equilibrium value) [33] and the calculation was carried out for the set of saturation times experimentally measured (0.5, 0.75, 1.0, 1.5, 2.0, 3.0, 4.0, 5.0 and 6.0 s). The theoretical STD values were compared to the experimental ones using the NOE R-factor [36,37] defined as:

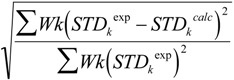
(1)


In this equation *STD_k_*^exp^ and *STD_k_^cale^* refer to experimental and calculated STD values for proton **k**.

## 4. Conclusions

The use of STD-NMR to the study of enzymatic systems has been problematic as they are frequently multiconformational systems with fast conversion rates, sort lived transition states and imbalanced concentration of species in the equilibrium. Thus, an enzymatic reaction optimized for the conversion of reactant into products is not, in principle, suitable for the study using STD-NMR. In the system used for this study, only electrons and hydride are transfer between reagents and products, therefore it can be better studied than other systems with larger structural changes. We have explored the application of STD-NMR to the non-natural system of FNR_ox_-NAD(P)^+^_ox_, that has been found to provide useful information into the mechanisms of the catalytic cycle.

We have found that the absolute fractional STD values exhibit a large dispersion between the diverse complexes and species that is canceled out when the relative STD or STD_0_ values were determined. Comparing them, most of the complexes afford similar results that are compatible with an extended binding along the central cleft where the nucleotides interact with their respective binding sub-sites. This has been observed in most of the solid state structures. In all cases the STD detected larger relative interactions for the adenosine nucleotide, in particular for adenine proton A2.

Interestingly, using this methodology we have been able to detect the interaction of NAD^+^ with FNR. The structure of the bound ligand was consistent with the binding of the adenosine nucleotide in its sub-binding pocket, while the nicotinamide moiety was folded back directed towards the bulk solution, far from the FNR as in the crystallographic structure 1quf obtained by soaking. This lack of interaction between the nicotinamide ring and the isoaloxacine moiety can explains lack of binging detection using UV measurements.

Attempts of perform STD quantification using CORCEMA-ST protocols have given good results for the case of the wild type FNR_ox_ with NADP^+^_ox_ based on the crystallographic structure with the same redox state cofactor and coenzyme. Within this pair the adenosine moiety predicted STD-NMR results coincide with the experimental, while for the nicotinamide ring some dispersion is observed. This reflects differences in small fluctuations within the binding site for both nucleotides. We attribute to the nicotinamide nucleotide a larger flexibility within the complex.

When we tried to use a snapshot extracted from MD simulations and the catalytic pair FNR_ox_-NADPH the STD quantification was far from the experimental results. This should be due to the fact that the snapshot used for the calculation corresponds to an instantaneous structure that is far from the average steady that is what finally experimentally observed and in this case this structure must be far from the average.

Then, we calculate a new structure for the same redox state than the crystallographic structure for the wild type for the Y303S mutant (FNR_ox_ and NADP^+^) using the MD structure and removing the hydride from the coenzyme and subjected to additional minimization. The STD calculated using CORCEMA-ST for the adenosine protons were consistent with the experimental values. Interestingly this was not the case for the nicotinamide ring, probably due to the larger flexibility of this half of the NADP^+^ within the complex, in particular for the case of the Y303S mutant. This mutation affects directly to the binding pocket of nicotinamide ring leaving even more freedom for motions inside the complex.

In summary, STD-NMR has proved to be a useful tool for the study of these complexes in particular when combined with additional data from other sources allowing quantification using CORCEMA-ST. We have detected two regions with different affinity for the enzyme corresponding to the two nucleotides. The nicotinamide moiety is less tightly bound into its binding pocket than the adenosine nucleotide that has a more rigid binding within the binding pocket.

## Supplementary Materials

Supplementary materials can be accessed at: http://www.mdpi.com/1420-3049/19/1/672/s1.
