# Supplementary material for: A STD-NMR Study of the Interaction of the Anabaena Ferredoxin-NADP+ Reductase with the Coenzyme
Source: Molecules. 2014 Jan 7;19(1):672–85. doi: 10.3390/molecules19010672 (PMC6272016; doi:10.3390/molecules19010672)

Supplementary Materials

Figure S1. FNR-NAD STD experiment (top), on-resonance (middle) and off-resonance (bottom).

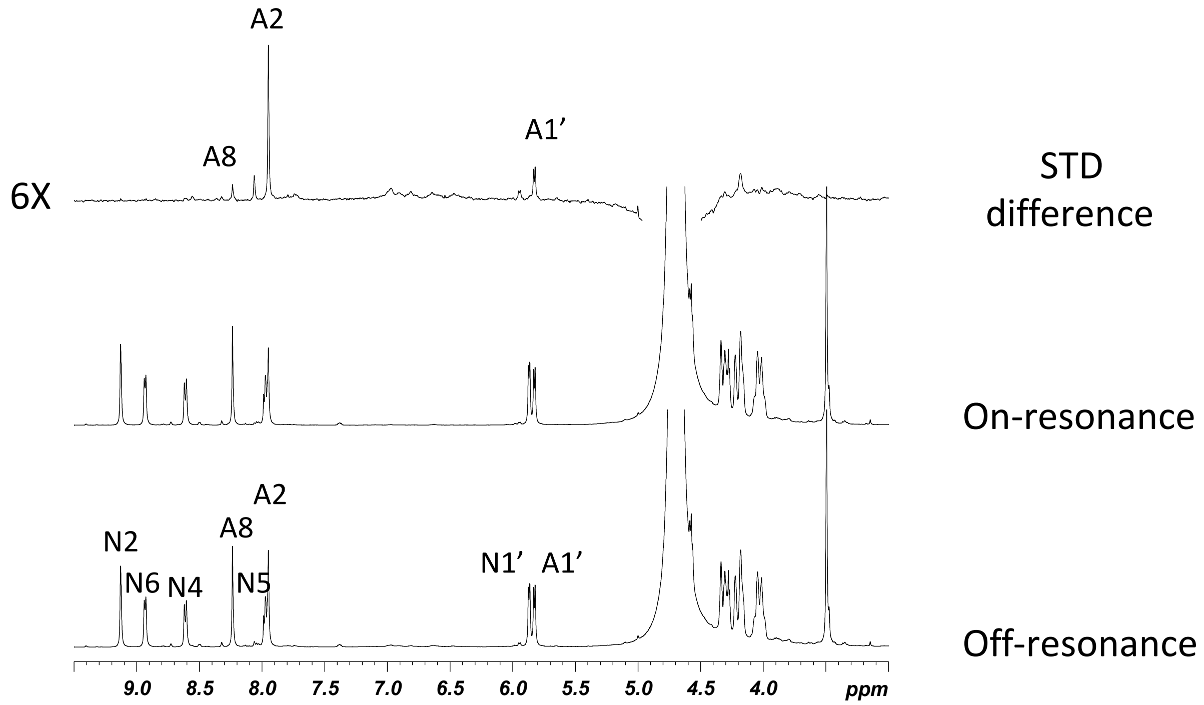

Figure S2. FNR-NADP STD experiment (top), on-resonance (middle) and off-resonance (bottom).

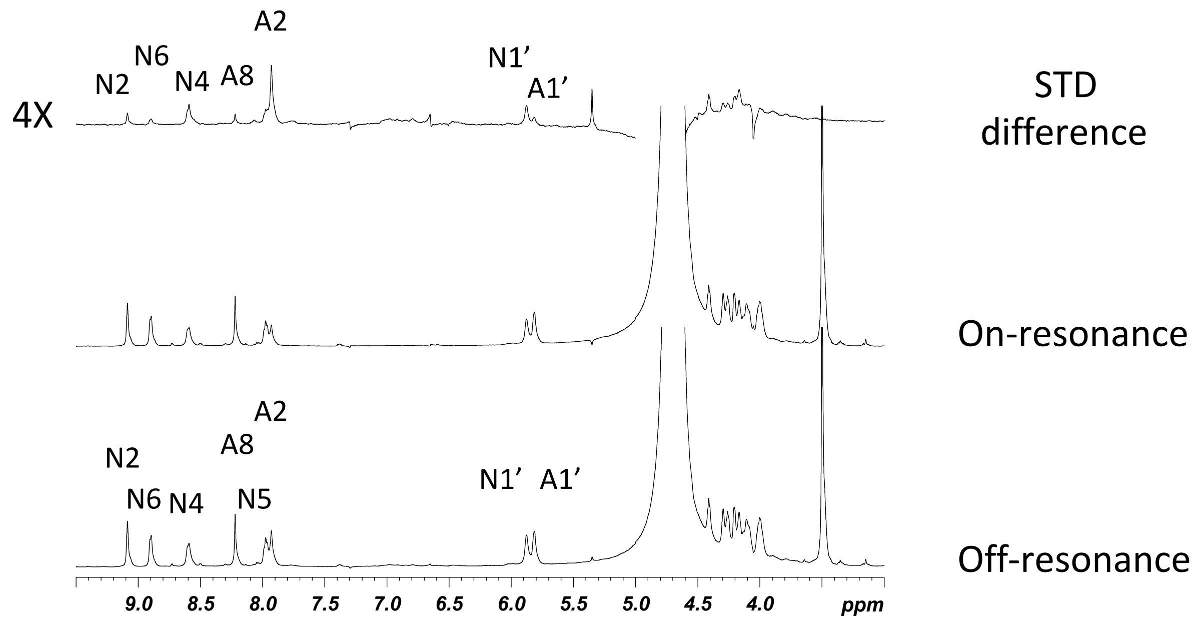

Supplement: Supplementary file 1 [file molecules-19-00672-s001.pdf]
